# Evaluation of the solitary pulmonary nodule: size matters, but do not ignore the power of morphology

**DOI:** 10.1007/s13244-017-0581-2

**Published:** 2017-11-15

**Authors:** Annemie Snoeckx, Pieter Reyntiens, Damien Desbuquoit, Maarten J. Spinhoven, Paul E. Van Schil, Jan P. van Meerbeeck, Paul M. Parizel

**Affiliations:** 1 Department of Radiology, Antwerp University Hospital & University of Antwerp, Wilrijkstraat 10, 2650 Edegem, Belgium; 2 Department of Thoracic and Vascular Surgery, Antwerp University Hospital & University of Antwerp, Wilrijkstraat 10, 2650 Edegem, Belgium; 3 Department of Pulmonary Medicine, Antwerp University Hospital & University of Antwerp, Wilrijkstraat 10, 2650 Edegem, Belgium

**Keywords:** Computed tomography, Solitary pulmonary nodule, Morphology, Lung cancer, Lung cancer screening

## Abstract

**Abstract:**

Subsequent to the widespread use of multidetector computed tomography and growing interest in lung cancer screening, small pulmonary nodules are more frequently detected. The differential diagnosis for a solitary pulmonary nodule is extremely broad and includes both benign and malignant causes. Recognition of early lung cancers is vital, since stage at diagnosis is crucial for prognosis. Estimation of the probability of malignancy is a challenging task, but crucial for follow-up and further work-up. In addition to the clinical setting and metabolic assessment, morphological assessment on thin-section computed tomography is essential. Size and growth are key factors in assessment of the malignant potential of a nodule. The likelihood of malignancy positively correlates with nodule diameter: as the diameter increases, so does the likelihood of malignancy. Although there is a considerable overlap in the features of benign and malignant nodules, the importance of morphology however should not be underestimated. Features that are associated with benignity include a perifissural location and triangular morphology, internal fat and benign calcifications. Malignancy is suspected in nodules presenting with spiculation, lobulation, pleural indentation, vascular convergence sign, associated cystic airspace, bubble-like lucencies, irregular air bronchogram, and subsolid morphology. Nodules often show different features and combination of findings is certainly more powerful.

***Teaching points*:**

• *Size of a pulmonary nodule is important, but morphological assessment should not be underestimated*.

• *Lung nodules should be evaluated on thin section CT, in both lung and mediastinal window setting*.

• *Features associated with benignity include a triangular morphology, internal fat and calcifications*.

• *Spiculation, pleural retraction and notch sign are highly suggestive of a malignant nature*.

• *Complex features (e.g. bubble-like lucencies) are highly indicative of a malignant nature*.

## Introduction

Subsequent to the widespread use of multidetector computed tomography (MDCT) and the growing interest in lung cancer screening, small pulmonary nodules are more frequently detected. Moreover, the global disease burden of lung cancer is on the rise [1]. A solitary pulmonary nodule (SPN) is defined as a rounded opacity in the lung, well or poorly defined, measuring up to 3 cm in diameter [2]. The differential diagnosis for SPNs is extremely broad, including both benign and malignant causes. Recognition of early lung cancers is vital since stage at diagnosis is crucial for prognosis. Estimation of the probability of malignancy is a diagnostic challenge, but is crucial for follow-up or further work-up. First step in this assessment is an evaluation of the clinical parameters such as signs and symptoms, patient age, smoking history, exposure, family history, associated lung diseases, and previous clinical history [3]. Second step is the imaging evaluation. Size, growth, and doubling time are key factors in assessing the malignant potential of a nodule. The likelihood of malignancy positively correlates with nodule diameter: as the diameter increases, so does the likelihood of malignancy. Malignancy, however, is not excluded in small nodules. Lack of growth does not always indicate benignity since adenocarcinomas (in particular those presenting as subsolid nodule) can be slow-growing tumours. Moreover some benign lesions, e.g. intrapulmonary lymph nodes, may show growth and have a volume doubling time in the range of malignant nodules [4]. Although imaging features of benign and malignant nodules show overlap, careful evaluation of morphologic features is an essential element of pulmonary nodule assessment. Nodule morphology should be evaluated on contiguous thin sections in axial, sagittal, and coronal planes. Investigation of nodule metabolism with 18F–fluorodeoxyglucose (FDG) positron emission tomography (PET) can have an additional value, but one needs to keep in mind that small nodules (< 8 mm), adenocarcinoma precursors and invasive adenocarcinomas with lepidic growth, as well as carcinoids can show low or no uptake [5]. In these lesions morphological assessment is crucial in order not to delay diagnosis. A recent study by Chung et al. [6] on a large set of subsolid nodules from lung cancer screening trials, showed that careful assessment of morphology in subsolid nodules could tremendously increase identification of malignant lesions. This result emphasises the importance of morphology as additional parameter to size and growth in regard to assessing likelihood of malignancy.

Several quantitative prediction models have been developed to assist in assessing the likelihood of malignancy. Different models exist for screen-detected nodules and nodules detected in non-screening populations, including models from Gurney [3, 7], the Mayo Clinic [8], Herder [9], Veterans Association [10], Peking University People’s Hospital (PKUPH) [11], Brock University [12], and Bayesian Malignancy Calculator by Soardi [13]. Whereas in more recent nodule calculators new features are taken into account (e.g. uptake on PET, contrast enhancement, volume doubling time), the number of morphologic features remains limited. Moreover variability among the features exists between different models. Likelihood of malignancy and odds ratios from these nodule calculators are summarised in Table 1.

**Table 1:** Likelihood and odds ratios for malignancy regarding morphological features in solitary pulmonary nodules

	Gurney et al. Radiology 1993 [3]	Swensen et al. Arch Intern Med 1997 [8]	Li et al. World J Surg 2012 [11]	McWilliams et al. N Engl J Med 2013 [12]	Soardi et al. Eur Radiol 2015 [13]
Population	Non-screen	Non-screen	Non-screen	Screen-detected	Non-screen
Number of nodules studied	Literature review	629	371	12,029	343
Morphological features					
Subsolid				No spiculation Ground glass: OR 0.74 (CI 0.40–1.35) Part-solid: OR 1.40 (CI 0.72–2.74) With Spiculation Ground glass: OR 0.88 (CI 0.48–1.62) Part-solid: OR 1.46 (CI 0.74–2.88)	
Smooth	LHR 0.30 (CI 0.20–0.41)		OR 0.245 (CI 0.133–0.451)		LHR 0.293 (smooth, elliptical, polygonal)
Lobulated	LHR 0.74 (CI 0.64–0.84)	OR 2.520 (CI 1.423–4.433)			LHR 0.735 (minimally lobulated) LHR 1.888 (deeply lobulated)
Spiculated	LHR 5.54 (CI 5.46–5.63)	OR 5.789 (CI 3.332–10.057)	OR 2.088 (CI 1.055–4.135)	OR 2.17 (CI 1.16–4.05)	LHR 7.884
Calcification	LHR 0.01 (CI 0–0.03)		OR 0.199 (CI 0.067–0.587)		
Cavitation		OR 3.05 (CI 1.078–8.646)			

*CI = 95% Confidence Interval, LHR likelihood ratio, OR odds ratio*

This pictorial review focuses on the morphologic evaluation of the solitary pulmonary nodule, with a 5-step approach and evaluation of 15 features (Table 2). Although these morphologic features are discussed one at a time, in practice a single nodule can show a variety of different features and combination of features is often even more powerful.

**Table 2:** Step-wise approach for morphological assessment of the solitary pulmonary nodule

Density	1. Solid 2. Subsolid
Shape	3. Round or oval 4. Triangular or polygonal
Margins	5. Smooth 6. Lobulated 7. Spiculated
Internal characteristics	8. Fat 9. Calcification 10. Cavitation
Complex findings	11. Pleural retraction 12. Air bronchogram 13. Bubble like lucencies 14. Cystic Airspace 15. Vascular convergence

## Morphologic features

### Density

**Fig. 1:**
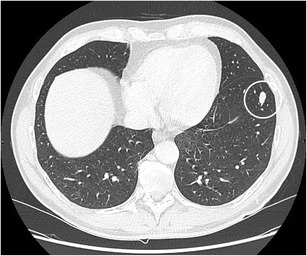
Axial chest CT scan, lung window setting, in a 54-year-old man with persistent cough shows a sharply delineated oval nodule in the left lower lobe. Since the lesion slowly increased in size, lobectomy was performed. Histopathology showed a 1 cm typical carcinoid

**Fig. 2:**
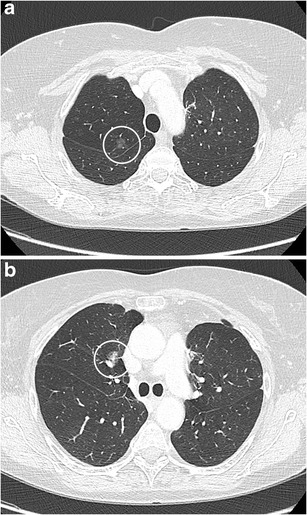
A 63-year-old woman with a previous history of lobectomy of the right upper lobe (10 years earlier) for invasive adenocarcinoma and wedge excision of the left upper lobe (2 years earlier) for minimally invasive adenocarcinoma was referred for CT. This follow-up chest CT showed numerous subsolid nodules in both lungs, with two part-solid lesions in the right middle lobe, increasing in size and density. Axial CT-image in lung window setting shows a pure ground glass nodule in the apex (Fig. 2a) and more centrally located part-solid nodule (Fig. 2b) with both ground-glass and dense component. Histopathological examination after lobectomy showed an adenocarcinoma in situ in the pure ground glass lesion and minimally invasive adenocarcinoma in the subsolid lesion

**Fig. 3:**
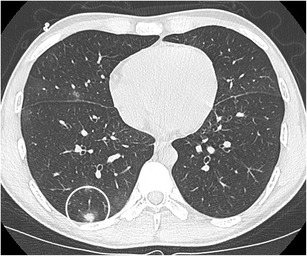
Axial CT-image in lung window setting in a 25-year-old immunocompromised man with a myelodysplastic syndrome treated with induction chemotherapy shows a subpleural nodule in the right lower lobe. The nodule has a predominant solid aspect with surrounding ground glass halo. Also note the small foci of ground glass in the right lung. Clinical and laboratory findings were consistent with invasive aspergillosis

A solitary pulmonary nodule (SPN) is defined as a rounded opacity, well or poorly defined, measuring up to 3 cm in diameter. The first step in assessment is defining nodule attenuation: solid or subsolid. Solid means that the density of the nodule obscures the underlying parenchyma (Fig. 1). Subsolid nodules contain a proportion of ground glass and are divided into pure ground glass nodules (Fig. 2a) and part-solid nodules (Fig. 2b). The density of ground glass is higher than that of normal lung parenchyma, but the normal lung architecture is preserved with normal bronchial and vascular margins [2]. The ground-glass component surrounding solid nodules is also referred to as “halo-sign” [2]. In a patient with neutropenia, finding such a nodule is highly suggestive of aspergillus infection (Fig. 3). A halo sign can also be seen in other benign conditions such as eosinophilic pneumonia, organising pneumonia, tuberculosis, cytomegalovirus, herpes simplex virus. In malignant nodules the halo sign is caused by local tumour spread or so-called lepidic growth pattern in which tumour cells proliferate along the surface of intact alveolar walls without stromal or vascular invasion [14]. In contrast to common infectious causes, these nodules typically persist during follow-up. According to the new World Health Organisation classification, these part-solid nodules correspond to minimally invasive adenocarcinoma (MIA) and lepidic predominant adenocarcinoma (LPA) [15]. Recognition of a subsolid morphology is crucial, since part-solid nodules have a significantly higher risk of malignancy compared to solid nodules [16, 17]. Change in morphology of these nodules, rather than size, suggests increase in invasiveness [18]. The reverse halo sign (ground glass surrounded by a ring of consolidation) can be found in cryptogenic organising pneumonia or in lung cancer nodules after radiofrequency ablation [19].

### Shape

**Fig. 4:**
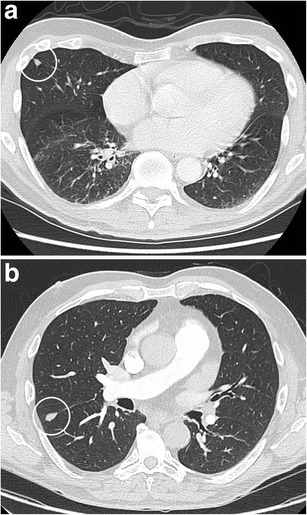
Two well-delineated intrapulmonary lymph nodules with triangular morphology. **a** In a 72-year-old man with neurological diplopia and equilibrium disturbances chest CT was performed to rule out a paraneoplastic cause. Axial CT-image in lung window setting shows a subpleural triangular nodule. The nodule has the typical morphology of an intrapulmonary lymph node. Also note the discrete thin septal lines connecting the lesion to the pleura Because of the benign nature, diagnosis was not confirmed on histopathology. Follow-up CT showed no change in size or morphology. **b** Incidental finding of a nodule peripherally located in the right lower lobe with clear triangular morphology. These findings are virtually pathognomonic for an intrapulmonary lymph node

**Fig. 5:**
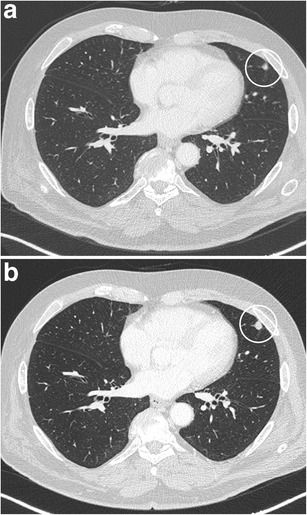
Axial CT-images, lung window setting, in a 67-year-old man with a previous history of melanoma show a small solid subpleural nodule in the lingula (**a**). The lesion has a solid, round, relatively smooth morphology. Since the lesion had doubled in volume over 1 year time (**b**), robot-assisted wedge-excision was performed. Histopathologic examination showed findings consistent with an intrapulmonary lymph node and showed no arguments for malignancy or melanoma metastasis

The typical shape of a SPN is round or oval. A solid nodule that is completely round has a lower likelihood of malignancy compared with solid nodules with a more complex shape. In contrast, a subsolid nodule with a round shape is more likely to be malignant [20]. Nowadays there is increased awareness for perifissural nodules (PFNs), which most commonly correspond to intrapulmonary lymph nodes. These intrapulmonary lymph nodes are fissure attached solid nodules, generally with a smooth margin, triangular or polygonal shape, oval or lentiform morphology. They usually lie within 15 mm of a pleural surface (Fig. 4) [21]. Atypical PFNs are nodules with the same typical triangular shape where the fissure is not clearly visible or lesions with a convex morphology on one side and rounded on the other. Intrapulmonary lymph nodes often have one or more septal lines connecting them to the pleura. In particular, these nodules warrant evaluation on thin section CT-images with reconstruction in different planes to assess the relationship with the fissure and to demonstrate the more flat shape. PFNs with spiculated morphology or crossing a fissure should not be classified as benign and warrant further work-up [4, 22]. Whereas growth is an indicator of malignancy, it is known that intrapulmonary lymph nodes can have the same volume doubling times as malignant nodules (Fig. 5) [23]. Intrapulmonary lymph nodes are common in daily practice. Data from lung cancer screening studies show that up to 28% of detected nodules correspond to PFNs, with no malignancies found in typical and atypical PFNs [4, 24]. Recognising intrapulmonary lymph nodes is crucial to prevent unnecessary controls or aggressive interventions.

### Margins

#### Smooth margin

**Fig. 6:**
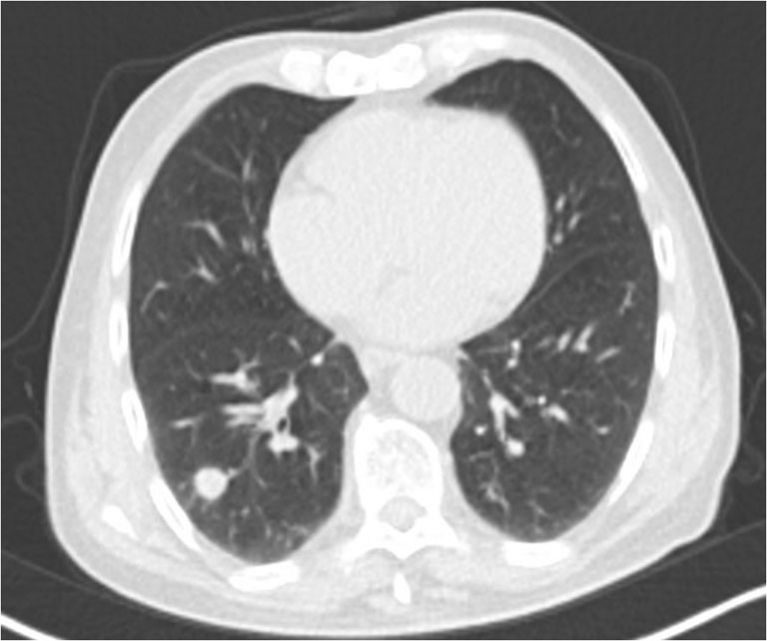
A 79-year-old man with a previous history of colon carcinoma with lymph node metastases 4 years earlier presented during follow-up with a 15 mm solitary pulmonary nodule. Axial CT in lung window setting shows a 15 mm round well-delineated solid nodule with smooth border in the right lower lobe. Histopathological examination after robot-assisted lobectomy showed a moderately differentiated squamous cell carcinoma

A smooth margin is generally associated with benignity. But although it is more common in benign solitary pulmonary nodules, it does not exclude malignancy (Fig. 6). About 21% up to one third of malignant SPNs have smooth margins [5, 25].

#### Lobulation

**Fig. 7:**
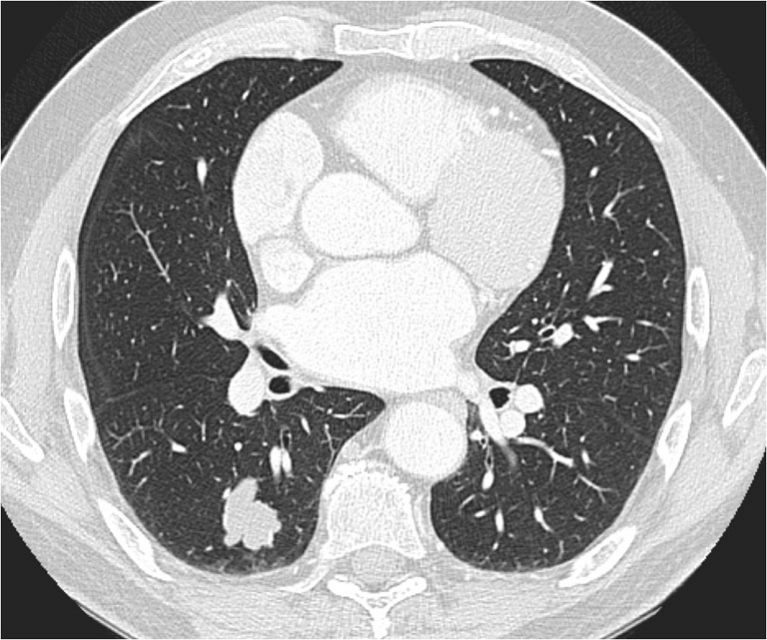
Axial CT-image in a 72-year-old man who presented with persistent cough, shows a well-delineated nodule in the right lower lobe with prominent lobulated appearance. Histopathology showed an invasive adenocarcinoma

**Fig. 8:**
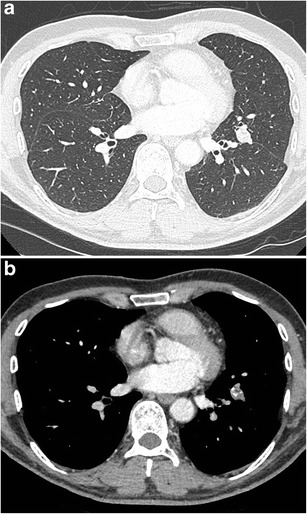
Incidental finding of a solitary pulmonary nodule in a 50-year-old man. Axial CT-image in lung window setting **a** shows a well-delineated nodule with clear lobulated morphology in the left upper lobe. The absence of growth, internal benign-looking calcifications and clear hypodense areas (corresponding to fat) on the images in mediastinal window setting **b** led to the probable diagnosis of a hamartoma

**Fig. 9:**
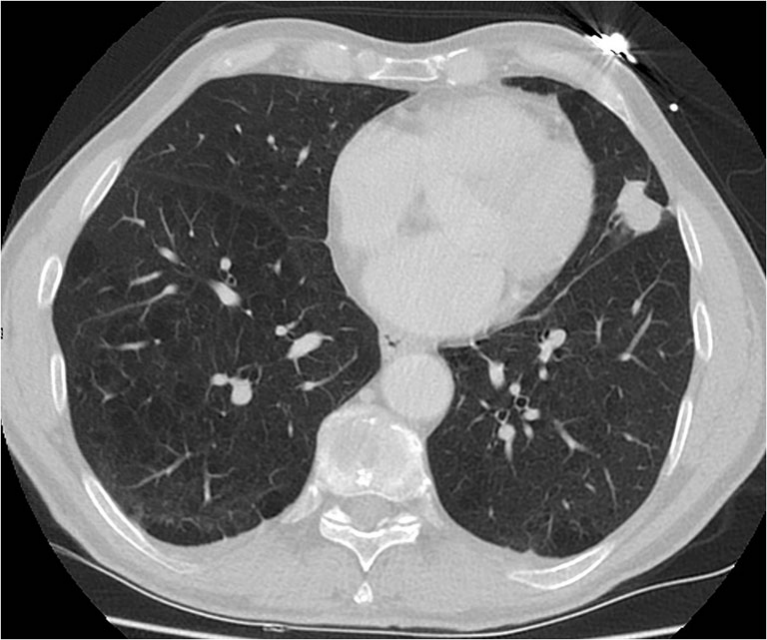
78-year-old man with a previous history of achilles tendon rupture and slight postoperative dyspnoea. Chest radiograph (not shown) showed a nodule in the left lung. Axial CT scan in lung window setting shows a 28 mm large, well-delineated nodule in the lingula with prominent indentation of the contour (notch). Histopathologic examination after lobectomy showed a large cell neuroendocrine carcinoma

**Fig. 10:**
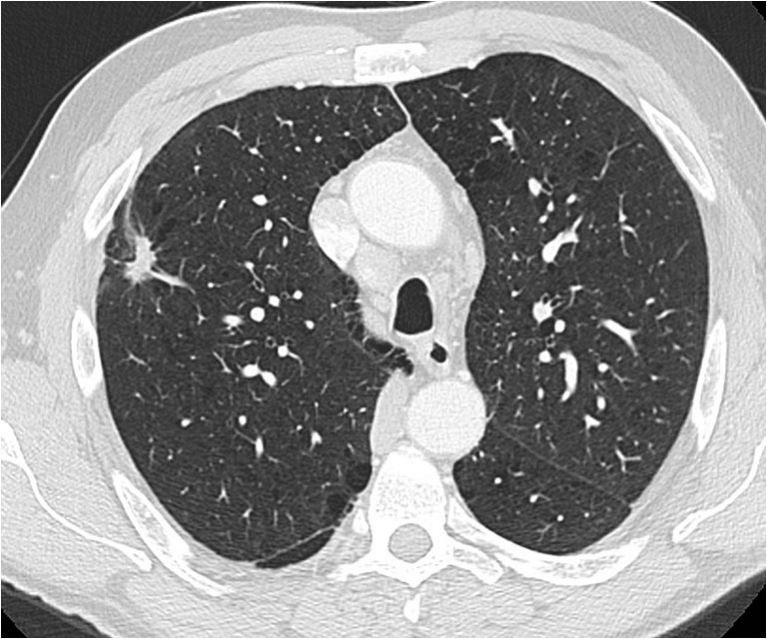
Incidental finding in a 61-year-old heavy smoker of a 15 mm large nodule in the right upper lobe with spiculation and a clear notch sign. Also note the enlarged paratracheal lymph node. Histopathology showed a small cell lung cancer

Lobulation in a nodule is attributed to different or uneven growth rates, a finding that is highly associated with malignancy (Fig. 7) [3]. In part-solid nodules a lobulated border suggests invasiveness [26, 27]. The finding is not uncommon in carcinoids [28]. Benign lobulation is the result of hyperplasia of adjacent connective tissue and cicatricial contraction. The margin should be carefully evaluated on thin slice CT-images to differentiate it with satellite micronodules. In benign nodules lobulation is often seen in hamartomas (Fig. 8) [29]. A particular form of lobulation is the “notch sign”. A notch is defined as an abrupt bulging of the lesion contour. This finding is relatively frequent in malignant nodules (Figs. 9 and 10), but can also be seen in benign conditions such as granulomatous diseases [30, 31].

#### Spiculation

**Fig. 11:**
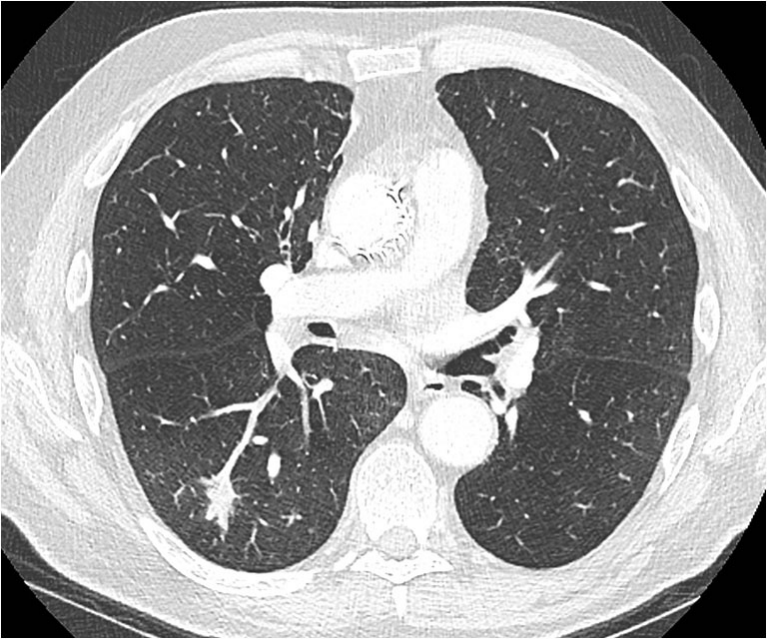
A 75-year-old patient with a previous history of squamous cell carcinoma of the right aryepiglottic fold presented during follow-up with a solitary 2 cm nodule in the right lower lobe. The head-and-neck tumour was treated 8 years before with curative intent. At the time of diagnosis of the SPN, the patient was also diagnosed with a colon carcinoma (adenocarcinoma). Axial CT-image shows an obviously spiculated nodule in the right lower lobe: these findings are suspicious for a second primary rather than solitary metastasis. Histopathologic examination showed a squamous cell carcinoma, with different growth pattern compared to the initial aryepiglottic tumour, making a third primary more likely

**Fig. 12:**
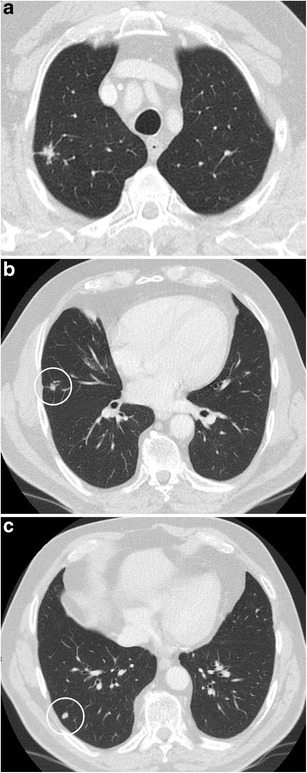
Incidental finding of a pulmonary nodule in a 72-year-old man with thoracic pain in whom the initial chest CT was performed to rule out pulmonary embolism. Three lesions were found: **a** a 15 mm nodule with moderate uptake on 18F–FDG-PET in the right upper lobe, **b** a 9 mm nodule with no uptake in the right middle lobe and **c** a triangular nodule in the right lower lobe with no uptake on PET. Both lesions in the right upper and middle lobe showed a suspicious spiculated morphology. The lesion in the right lower lobe was thought to be an intrapulmonary lymph node because of the morphology and location. Although the multidisciplinary thoracic oncology tumour board was convinced that this nodule was probably benign, the board decided to recommend resection (with a small wedge excision) to rule out malignancy with certainty since the other two lesions looked both suspicious. Histopathologic examination after wedge excision of the three nodules showed acinar type adenocarcinoma in the largest lesion, adenocarcinoma with some lepidic growth (which explains why PET was negative in this lesion) in the smallest spiculated lesion and confirmed diagnosis of an intrapulmonary lymph node in the right lower lobe

**Fig. 13:**
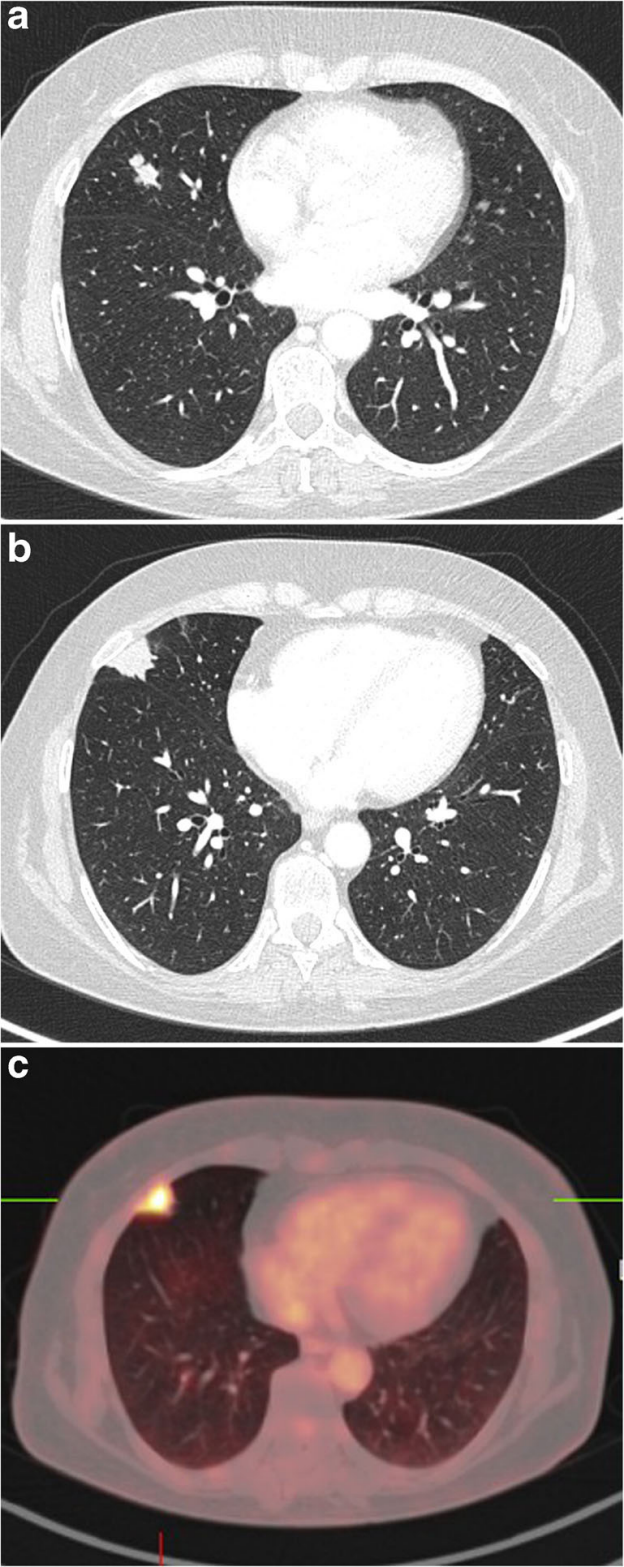
A 54-year-old woman with a previous history of brain metastases from a small cell lung carcinoma, presented during follow-up with two lung nodules. Axial CT in lung window setting shows two nodules in the right middle lobe: a 17 mm spiculated and lobulated nodule (**a**) that only showed minor uptake on 18F-FDG-PET and a spiculated 22 mm pleural based nodule (**b**) with very intense uptake (**c**). The smallest nodule had been described in previous reports. The largest spiculated peripheral nodule was a new finding. Because of the high clinical suspicion of tumour recurrence and absence of extrathoracic disease on 18F-FDG-PET, thoracoscopic wedge excision of both nodules was performed. Histopathologic examination of the smallest spiculated nodule showed findings consistent with a typical carcinoid. The larger spiculated subpleural lesion was a tuberculoma. There were no signs on histopathology for small cell lung cancer recurrence. Even if imaging shows suspicious findings, new lesions in oncology patients are not always tumour recurrence. Depending on the clinical situation, histopathological proof may be mandatory in these cases

Spiculation (also called sunburst or corona radiata sign) is caused by interlobular septal thickening, fibrosis caused by obstruction of pulmonary vessels or lymphatic channels filled with tumour cells [30]. It is highly predictive of malignancy with a positive predictive value up to 90% [25]. A nodule with a spicular margin (Figs. 11 and 12) is much more likely to be malignant than one with a smooth, well-defined edge [3, 32]. Data from the Dutch-Belgian randomised lung cancer screening trial (NELSON) show that spiculation (as well as lobulation) has an increased likelihood for lung cancer when compared to smooth, round, or polygonal shape. Benign conditions that can manifest as spiculated nodule are infection, tuberculomas, inflammatory pseudotumours, focal atelectasis, and fibrosis (Fig. 13). In subsolid nodules, spiculation is a predictive feature for invasiveness [27].

### Internal characteristics

#### Fat attenuation

**Fig. 14:**
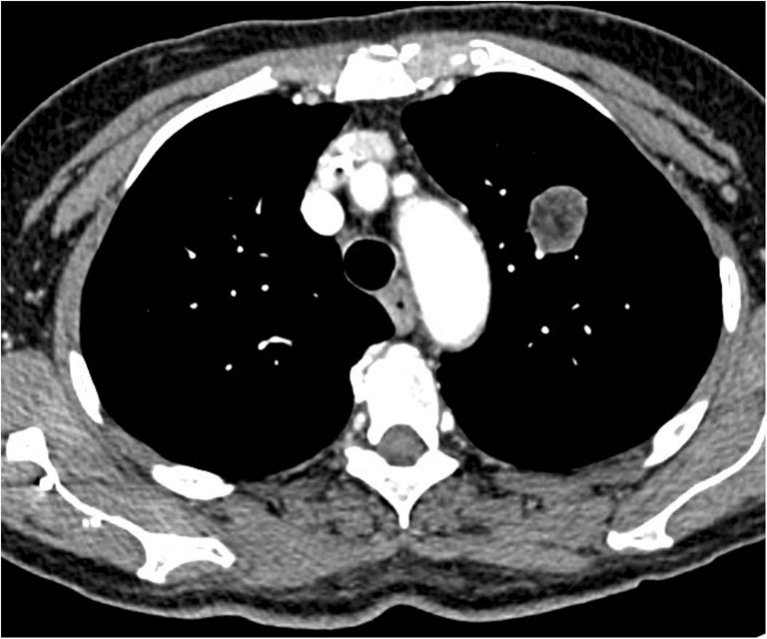
Axial chest CT scan, mediastinal window setting, in a 69-year-old woman with a previous history of gallbladder carcinoma. Because of increase in tumour markers, an 18F-FDG-PET-CT was ordered, showing a well delineated round nodule with absence of 18F-FDG-uptake. The internal aspect of the nodule shows small hypodense foci with negative Houndsfield units on measurement. The typical morphology, absence of uptake on 18F-FDG-PET and absence of evolution are consistent with a benign hamartoma

**Fig. 15:**
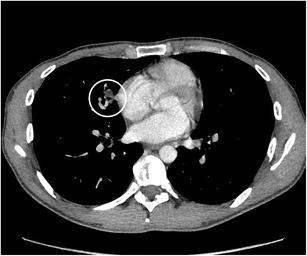
Axial CT-image in mediastinal window setting in a 33-year-old fire-eater shows a well delineated nodule in the right middle lobe with internal fat component. Findings are consistent with small lipoid pneumonia. The nodule completely resolved after treatment

Appraisal of the internal characteristics of SPNs requires evaluation of the nodule in soft tissue and bone window. Intranodular fat typically has a CT-attenuation of −40 to −120 Hounsfield Units. The presence of fat is a reliable indicator of hamartoma (Fig. 14) although only 50% of hamartomas contain significant fat deposits [29]. When other morphologic characteristics suggest a hamartoma, but no fat can be measured on CT, magnetic resonance imaging with chemical-shift sequences can be considered [33]. Differential diagnosis of a fat containing pulmonary nodule includes metastasis from liposarcoma or renal cell cancer and lipoid pneumonia (Fig. 15).

#### Calcification

**Fig. 16:**
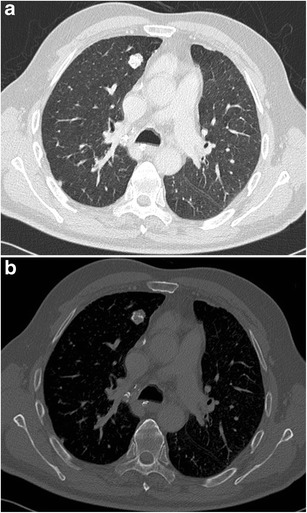
Incidental finding in a 75-year-old man. Axial CT-image in lung window setting **a** shows a well delineated somewhat lobulated nodule in the right upper lobe. In bone window **b** the chunky popcorn calcifications can easily be appreciated. Findings are consistent with a hamartoma

**Fig. 17:**
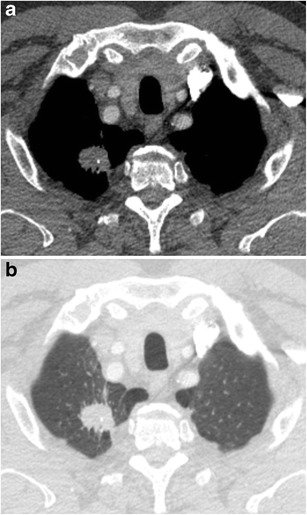
Axial CT-images in lung (**a**) and mediastinal (**b**) window setting show a 2.4 cm nodule in the apex of the right upper lobe. The lesion is somewhat spiculated with scarse small dot-like calcifications. Although postinfectious scarring is common in the lung apices, there are no abnormalities on the left. Moreover the lesion is relatively round and the calcifications do not have a typical benign nature. Histopathologic examination after lobectomy showed a moderately differentiated squamous cell carcinoma

Calcification in a nodule is generally associated with benign conditions. Whereas calcifications are not uncommon in lung cancer (occurring up to 13%), they are uncommon (2%) in lung cancer presenting as SPN [34]. Calcification morphology is crucial for assessing the likelihood of benignity/malignancy. Benign calcifications are central, diffuse solid, or laminated and associated with prior infections such as histoplasmosis and tuberculosis. “Popcorn-like” calcifications are characteristic of chondroid calcifications in hamartomas (Fig. 16). Dense, uniform calcifications are frequently encountered and strongly related with benignity [35]. In malignant nodules, dystrophic calcifications (Fig. 17) are more diffuse, amorphous or punctate, few in number and more eccentric in location [3, 36]. Punctate calcifications may also occur in lung cancer, due to engulfment of a pre-existing calcified granulomatous lesion, as well as in metastases [5]. Special attention should be payed to scar-like lesions, often found in the lung apices, since these lesions can harbour lung scar cancers [37]. Calcification in apical lesions is generally regarded as benign, often sequelae of tuberculosis. Regular follow-up is warranted especially when lesions are new, asymmetric and growing [38]. Calcification in carcinoids can occur in up to one-third of tumours and is more common in central carcinoids [28, 39].

#### Cavitation

**Fig. 18:**
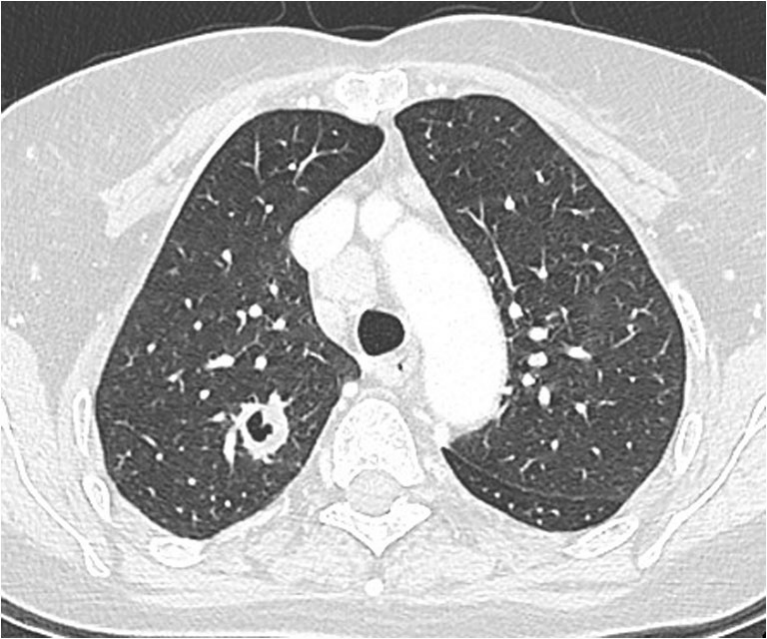
Chest radiograph in a 59-year-old woman with a previous history of heart transplantation showed a nodule in the right upper lobe. This was confirmed on chest CT scan with axial images in lung window setting showing a 2.4 cm nodule with central cavitation. Since efforts to prove an infectious cause remained unsuccessful and the lesion persisted, wedge excision was performed. Histopathologic examination could not reveal any malignancy, but showed findings consistent with an aspergilloma

**Fig. 19:**
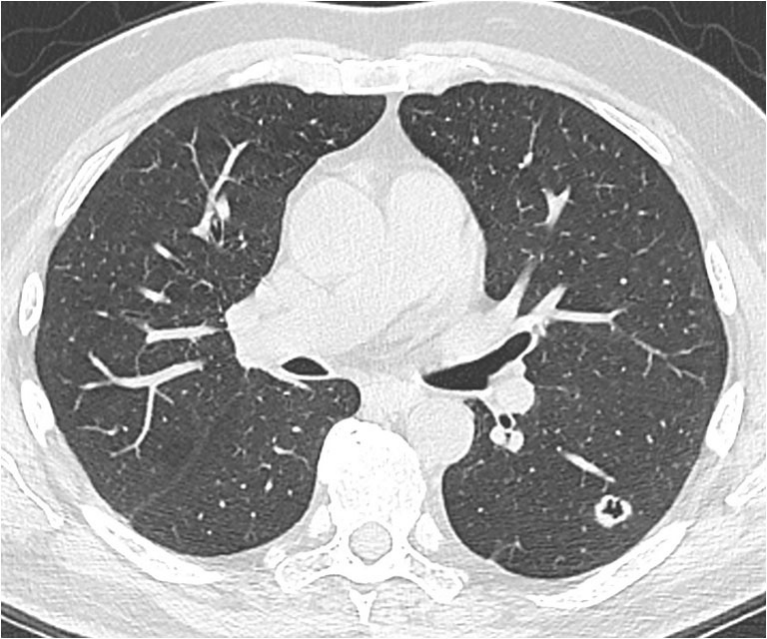
Chest CT in a 57-year-old man who presented with chronic cough. Axial CT-image in lung window setting shows a 12 mm well-defined round nodule in the left lower lobe. The nodule shows a central area of cavitation. Since there were clinically no arguments for an infectious cause, video-assisted thoracoscopic lobectomy was performed. Histopathologic examination showed a small squamous cell carcinoma

A cavity is defined as a gas-filled space, seen as lucency or low-attenuation area within pulmonary consolidation, mass or nodule [2]. Cavitation can occur in both benign and malignant nodules. In benign nodules, cavitation is mainly associated with abscesses (bacterial) pulmonary tuberculosis, histoplasmosis, aspergilloma (Fig. 18), and other fungal infections, Wegener granulomatosis, Churg-Strauss syndrome, rheumatoid arthritis. [40–42]. Even in infectious causes, there are often no clinical symptoms of infection, raising difficulties for diagnosis. Cavitation in malignant SPNs is caused by necrosis of the central portion and is mainly seen in squamous cell carcinoma (Fig. 19) and metastasis. Wall thickness, irregularity, and cavity morphology have been regarded as partially useful for differentiating benignity from malignancy, with great overlap of features [41, 43]. A thicker irregular wall and irregular inner contour are both observed in malignant nodules and in benign diseases. Associated ground glass, consolidation, bronchial wall thickening, and the presence of satellite nodules are indicative of a benign nature of the cavitary nodule. Since infectious (bacterial) or inflammatory cavitation often present with rapid changes, short-term follow-up with chest radiographs can be the key to diagnosis. Rapid progression in these cases excludes a malignant cause. Correlation with clinical setting and often histology is mandatory in cavitated nodules since imaging can barely differentiate between benign and malignant.

### Complex findings

#### Pleural retraction

**Fig. 20:**
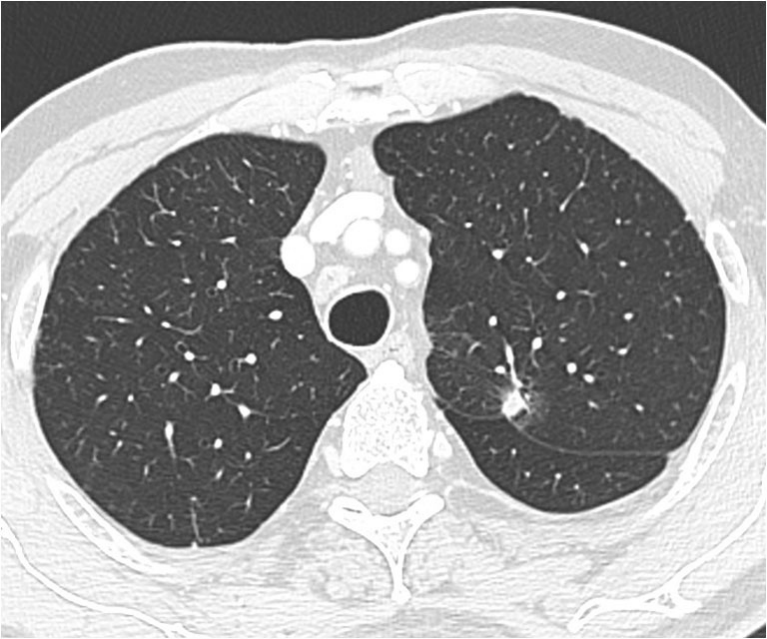
A 78-year-old man presented to the neurologist with symptoms of sensorimotor polyneuropathy. Chest CT-scan was performed to rule out a paraneoplastic cause. Axial CT-image in lung window setting shows an 8 mm nodule, relatively well-delineated with some discrete spiculation and ground glass component surrounding. Although the lesion is small, there is a prominent retraction of the adjacent fissure. Histopathologic examination proved the malignant nature, showing an adenocarcinoma

**Fig. 21:**
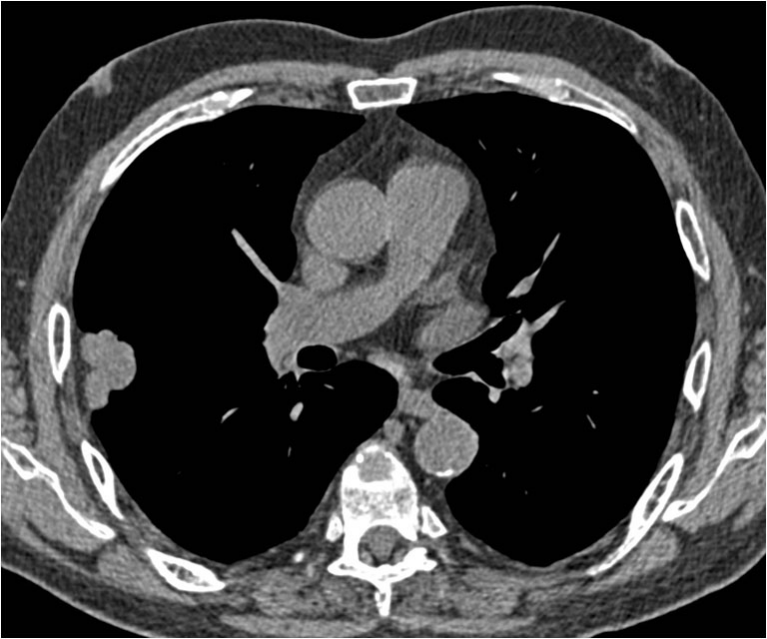
Chest CT in 62-year-old man with pancytopenia was performed to rule out infection. As an incidental finding a large lobulated subpleural nodule was noted. The axial CT-image in mediastinal window setting shows a triangular fat component, extending from the pleura into the lesion and corresponding to pleural retraction. Histopathology confirmed a malignant aetiology, with a poorly differentiated adenocarcinoma

**Fig. 22:**
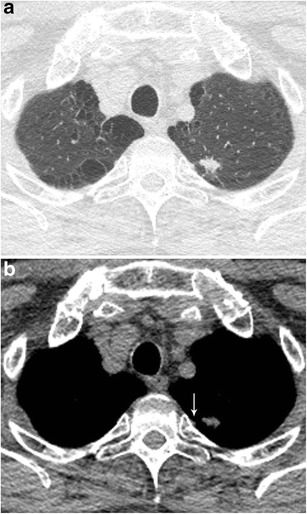
8F-FDG-PET-CT was performed in a 58-year-old man with unexplained fatigue and weight loss. PET (not shown) showed a lesion in the left apex with moderate uptake, corresponding on CT with a 1.2 cm spiculated nodule. On the axial CT-image in lung window setting **a** some fine linear strands extending to the pleura (pleural tags) are noted. In mediastinal window setting **b** a small triangular focus of retracted fat can be seen (white arrow). The nodule exhibits some fine linear strands or pleural tags. Histopathologic examination after lobectomy showed an adenocarcinoma with lepidic and acinar growth with focal invasion to the visceral pleural surface (PL2)

Pleural retraction represents pulling of the visceral pleura towards the pulmonary nodule. It is far more common in malignant (Fig. 20) than in benign SPNs [30, 32, 44]. The finding is rare in metastases or carcinoid tumours. On histopathology pleural retraction represents fibrotic bands [30]. In peripheral nodules in contact with the pleura, pleural retraction is often better visualised in mediastinal window (Fig. 21). Pleural retraction in a pure ground glass nodule is a predictive factor for invasiveness. These lesions are more likely to present invasive adenocarcinoma with lepidic growth and are less likely to be minimally invasive adenocarcinoma or adenocarcinoma in situ [27, 45, 46]. Pleural tags are defined as linear strands that extend from the nodule surface to the pleural surface. They correlate with thickening of the interlobular septa of the lung and can be caused by oedema, tumour extension, inflammation, or fibrosis. Pleural tags are common in malignant lesions [30, 47]. In a nodule not abutting the pleura, a pleural tag with soft-tissue component at the pleural end (to evaluate in mediastinal window) suggests visceral pleural invasion [47] (Fig. 22).

#### Air bronchogram

**Fig. 23:**
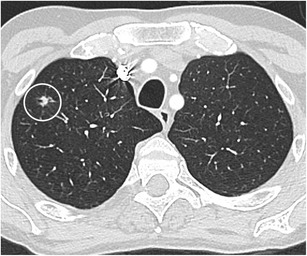
Incidental finding in a 48-year-old man. Axial CT in lung window setting shows a small round somewhat spiculated 7 mm nodule in the right lower lobe. The nodule clearly shows a bronchial interruption sign with abrupt cut-off of the bronchus running towards the nodule. Morphological findings are highly suspicious for a small primary lung cancer. 18F-FDG-PET showed moderate uptake in the nodule as well as in the left adrenal gland. Histopathologic examination of the adrenal gland showed a non-small cell lung cancer metastasis

**Fig. 24:**
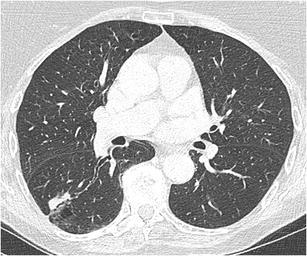
In a 74-year-old woman with persistent cough a 2.9 cm nodule was found on a chest radiograph. Chest CT was performed, with axial images in lung window setting showing an oval nodule in the right lower lobe. The nodule shows a prominent air bronchogram with irregular aspect, making the nodule suspicious for malignancy. Video-assisted thoracoscopic lobectomy was performed and a malignant cause (adenocarcinoma) was confirmed on histopathology

Air bronchogram is defined as a pattern of air-filled bronchi on a background of airless lung [2]. This used to be associated with infectious causes of consolidation and, therefore, “benignity”. However in the setting of a SPN, an air bronchogram is actually more frequent in malignant than in benign nodules [48, 49]. Qiang et al. [50] studied the tumour-bronchus relationship and described five types. In “Type 1” the bronchial lumen is patent up to the tumour. In “Type 2” the bronchus is contained in the tumour (Fig. 23). These types are more common in malignant nodules. A compressed and narrowed bronchus is defined as “Type 3”, and can occur in both benign and malignant nodules. Narrowing of the proximal bronchial tree is described as “type 4” and is associated with malignancy. “Type 5” is a bronchus compressed and flattened by the nodule with intact smooth wall. This type is mainly seen in benign nodules. Keeping in mind how a tumour with lepidic growth expands, it is not surprising that the air bronchogram in these tumours is smooth [32]. In contradistinction, a desmoplastic response may cause irregularities of the bronchogram (Fig. 24) [51]. When retraction of tumoural fibrosis occur, the air bronchogram can even become somewhat dilated. Although this sign can occur in all lung cancer cell types, it is more common in adenocarcinoma [48, 50, 52]. Studies suggest the association of this sign with an activated Epidermal Growth Factor Receptor (EGFR) mutation [53–55].

#### Bubble-like lucencies

**Fig. 25:**
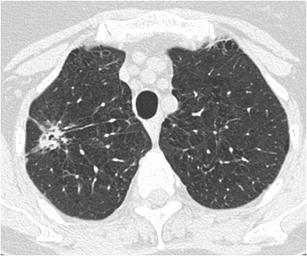
Complex lesion in the right upper lobe in a 61-year heavy smoker. Axial CT-image in lung window setting shows a 2.6 cm lesion with spiculated morphology, pleural tags and centrally small lucent foci, corresponding to the so-called “bubble-like-lucencies”. In the periphery of the lesion there are some small foci of ground glass appearance. Histopathologic examination after lobectomy showed a predominant acinar adenocarcinoma with areas of lepidic growth

**Fig. 26:**
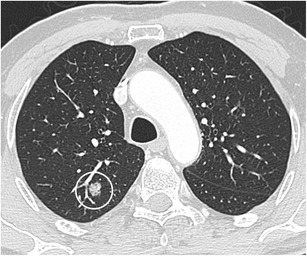
69-year-old man with recent diagnosis of a bladder carcinoma. 18F–FDG-PET was performed for staging and showed a well-delineated nodule in the right upper lobe. Axial CT-imaging in lung window setting show a heterogeneous aspect of the nodule with small hypodense foci or bubble-like-lucencies. Although the lesion did not show intense uptake on 18F-FDG-PET, lobectomy was performed based on the suspicious morphology. Histopathologic examination showed a 13 mm invasive adenocarcinoma with predominant lepidic growth

Bubble-like lucencies or “pseudocavitation” represents a sign different from the air bronchogram sign, which is branch-like. Bubble-like lucencies are areas of low attenuation due to small patent air containing bronchi in the nodule. This pattern is characteristic of replacement growth tumour on histopathology [56]. Other authors have also shown a relationship with the former bronchioloalveolar subtype of adenocarcinoma. The presence of these bubble-like lucencies is highly suggestive of malignancy (Figs. 25 and 26). When seen in subsolid nodules (in particular part-solid nodules), the finding is slightly more common in invasive adenocarcinomas than in preinvasive lesions [27]. It is uncommon in non-neoplastic nodules [30, 57].

#### Cystic airspace

**Fig. 27:**
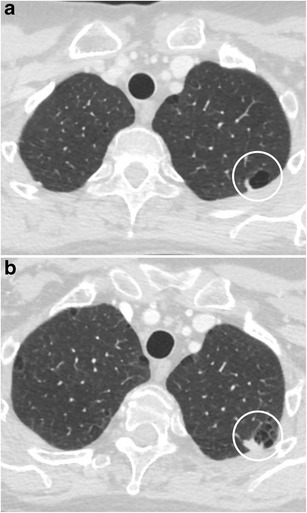
A 51-year-old woman with myelitis transversa presented on CT with a cystic airspace with mural nodule. Axial CT-image in lung window setting **a** shows a subpleural cystic airspace with a mural nodule with somewhat thin, bandlike morphology. Follow-up CT 6 months later **b** shows increase in size of the nodular component and more multicystic aspect of the cystic airspace. The lesion was found suspicious for a lung cancer associated with cystic airspaces. Lobectomy was performed and confirmed the malignant aetiology showing a poorly differentiated adenocarcinoma

A pulmonary nodule abutting the wall of a “cystic airspace” is rare, but more frequently encountered and recognised since lung cancer screening trials have been initiated. Data from the International Early Lung Cancer Action Program (I-ELCAP) show that 2% (13/595) of cancers at baseline and 12% (13/111) at annual screening showed this specific morphology [58]. Scholten et al. showed that 22.7% of the missed cancers in the NELSON lung cancer screening trial presented as a bulla with wall thickening [59]. This type of lesion is highly suspicious of malignancy. Adenocarcinoma is the most commonly encountered associated cancer, nevertheless squamous and small cell carcinomas can also be found. The mechanism of this cystic airspace formation is not yet fully unravelled. Current hypotheses on aetiologies as cause of the cystic airspace include a check-valve mechanism involving the small airways leading to outflow obstruction, lepidic growth of adenocarcinoma superimposed on destroyed alveolar walls, cystification of tumour and growth of adenocarcinoma along the wall of a preexisting bulla [58, 60, 61]. Association of a cystic airspace (Fig. 27) with nodule should alert the radiologist to the possibility of this entity in order not to delay diagnosis and treatment.

#### Vascular convergence

**Fig. 28:**
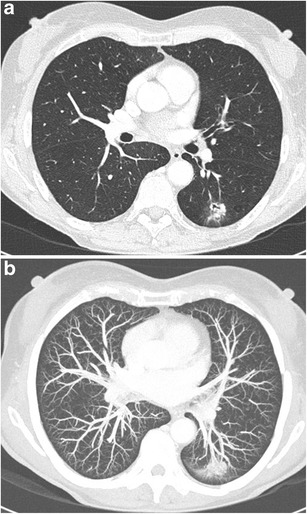
Cardiac CT in 64-year-old woman with chronic cough and cardiac complaints showed a nodule in the left lower lobe. Dedicated chest CT confirmed persistence of the nodule and solitary nature. Axial CT-images in lung window setting **a** show a complex nodule with spiculation, pleural tags, irregular air bronchogram with bronchial interruption sign and ground glass component. Maximum intensity projection (MIP) images **b** better demonstrate convergence of the vessels towards the lung nodule. Malignancy was confirmed after lobectomy with histopathologic examination showing a 2.1 cm invasive adenocarcinoma

The vascular convergence sign (Fig. 28) is described as vessels converging to a nodule without adjoining or contacting the edge of the nodule and is mainly seen in peripheral subsolid lung cancers. The rationale behind this sign is that angiogenesis is essential for tumour growth and metastasis. Hu et al. reported a malignancy rate of 85.2% in subsolid nodules expressing this sign. In their series it was also found in 40.0% of benign nodules [62].

## Conclusion

Assessing the likelihood of malignancy in pulmonary nodules remains a challenging task. Morphological assessment is only one part of the diagnostic puzzle, but its role should not be underestimated. A smooth border, triangular or polygonal shape with perifissural location, fat and popcorn calcifications indicate a benign nature. Features that suggest a malignant nature include a persistent subsolid morphology, spiculation, lobulation, and pleural retraction. More complex findings such as bronchial abnormalities, bubble-like lucencies, an associated cystic airspace and vascular convergence sign are also indicative of a high likelihood of malignancy. In subsolid nodules spiculation, lobulation, and pleural retraction are indicative for an invasive adenocarcinoma rather than a preinvasive lesion.
